# Vulnerability to watershed erosion and coastal deposition in the tropics

**DOI:** 10.1038/s41598-020-79402-y

**Published:** 2021-01-13

**Authors:** Trevor N. Browning, Derek E. Sawyer

**Affiliations:** School of Earth Sciences, The Ohio State University, 125 South Oval Mall, Columbus, OH 43214 USA

**Keywords:** Environmental impact, Geomorphology, Sedimentology

## Abstract

Over half of the global population is projected to live in the tropics by 2050. Sustainable land development will be challenged by enhanced sediment erosion and deposition, which can negatively impact water quality and ecosystem services in inland and coastal waterways. Existing erosion assessments treat watersheds and coastal zones separately, but we connect them in a two-part vulnerability index to watershed erosion and coastal deposition at 0.0004° (~ 45 m) resolution throughout the tropics. We use open-source datasets and a simple, GIS-based method geared toward tropical, novice end-users. Part 1 of the index reveals a majority of the tropics is vulnerable to erosion. Vulnerability is highest where there are co-occurrences of earthquakes, steep slopes, and high precipitation such as the Caribbean and Southeast Asia. In Part 2, we assess erosion vulnerability at 4 watersheds and include their coastal systems, which can enhance or diminish vulnerability of the entire system to coastal deposition.

## Introduction

The tropics are undergoing rapid economic and population growth as indicated by the highest economic growth rate over the past 30 years, and projections that indicate over half of the global population will live in the tropics by 2050^1^. The rapid growth will be accompanied by significant land use changes and associated soil erosion. Fast growth leads to rapid deforestation of undeveloped land for agricultural and mining operations, which displaces rural populations to urban areas and, in turn, increases urban expansion and land use change^1–4^. Borrelli, et al.^5^ demonstrate that the tropics currently have the highest potential erosion rates in the world correlated strongly to agricultural expansion and economic development within developing countries (75% of which are in the tropics^6^).

The tropics (± 23.5°) are prone to high erosion rates due to their consistently warm climate and prevalent rainfall both seasonally (higher latitudes) and year-round (near the equator)^1^. Many tropical areas are on active tectonic settings that are steep and mountainous (Caribbean islands, Southeast Asian coast and islands, etc.) leading to high weathering rates^7,8^. Watersheds in these areas can have short sediment transport pathways to the coast^9^ and short watershed soil residence times as a result of consistent rainfall^10,11^. In addition, extreme events, such as monsoons, hurricanes, and earthquake-induced landslides, are common in the tropics and among the most destructive on Earth^12^.

Importantly, land-derived soil erosion is not a localized problem, but rather has cascading effects downstream on water quality, ecosystems, and coastal zones. Fine-grained sediment and contaminants can cause microbial outbreaks and degrade water quality^13^, alter water chemistry^14^, and increase turbidity and suspended solid concentrations^15^. In many cases, topsoil in agricultural and developed areas is anthropogenically replenished in a persistent cycle, which increases sediment loads to streams and coasts halting bedrock weathering. Chemical weathering of bedrock is high in the tropics and consumes a large component of global CO_2_^7^.

Economically critical aquatic ecosystems (e.g. coral reefs)^16–18^ are at high risk to sedimentation (human and naturally induced) and the associated negative effects (mortality) that accompany it^19–21^. Along the coast, calcareous algae are reef builders^22^, seagrasses serve as fish breeding grounds^23^, and coral reef communities create diverse habitats^18^ and buffer the coast from wave action and storms^24^. Coastal seagrass communities are an important carbon capture and storage vehicle (~ 2 × more efficient than tropical rainforests per km^2^^25^). In freshwater ecosystems, sediment delivery and deposition degrades the quality of benthic habitats, and disrupts structural functions of freshwater ecosystems^26,27^.

Assessments of vulnerability to erosion tend to focus either on the watersheds on land or the coastal zone, without connecting them, and typically are at the regional or watershed scale. For example, Coastal Vulnerability Indices (CVIs) have been used extensively in recent years to assess either coastal erosion^28–30^ or watershed erosion^31–34^. Some focus on modeling potential watershed soil loss using the Revised Universal Soil Loss Equation (RUSLE) or other soil water erosion models, primarily at the watershed scale^35–37^ with few at a global scale^38,39^. Recent studies in India have focused on integrating risk indices^40,41^ for a more holistic approach by using multiple factors (physical, social, and geo-technical) to quantify risk to a region affected by erosion.

CVIs have not yet reached the global scale and center on identifying risk along the coastline while ignoring watershed activities such as land use. In contrast, RUSLE models focus on the potential soil loss in the watershed, but do not address sediment delivery to the coast or multiple types of land-use change. Despite recent improvements to RUSLE models^38,42^ there are still accuracy issues in developing countries and remote small land area regions (Southeast Asia, the Caribbean, and Pacific islands) where high resolution datasets are scarce or non-existent. Thus, less accurate datasets are utilized to form the critical underpinning parameters of RUSLE such as the rainfall erosivity factor^43^.

Our goal is to create a global index of the relative vulnerability of any watershed to erosion throughout the tropics and connect that to deposition in individual coastal zones using open-source datasets. Toward this end, we first created a simple, GIS-based, open-source Erosion Vulnerability Index (EVI). The EVI is a global risk index of all watersheds in the tropics, includes multiple land use change types, and is useful to map the relative vulnerability of any watershed to erosion throughout the tropics. Secondly, we create the Erosion and Deposition Vulnerability Index (EDVI), which combines the watershed erosion risk (EVI) with coastal deposition risk. While a tropics-wide EVI can be created in one process, the EDVI has to be calculated on an individual watershed. We demonstrate significant change between watershed EVI and the interconnected EDVI with 4 diverse case studies across the tropics (Burdekin River, Queensland, Australia; Mindanao River, Mindanao, Republic of the Philippines, Southeast Asia; Mana River, French Guiana, South America; Cavalla River, Liberia/Côte d’Ivoire/Guinea, Africa). All major regions of the tropics are represented including continents and islands. While the end product is high resolution (0.0004° × 0.0004° or ~ 45 × 45 m), many datasets lack coverage over smaller islands, especially in the Pacific Ocean and eastern Caribbean Sea. This simple GIS method relies solely on open-source data, which may allow groups in developing nations, government, industry, or research sectors better understand the erosion and deposition cycle in their region of interest.

## Results

### Tropics-wide EVI

**Figure 1:**
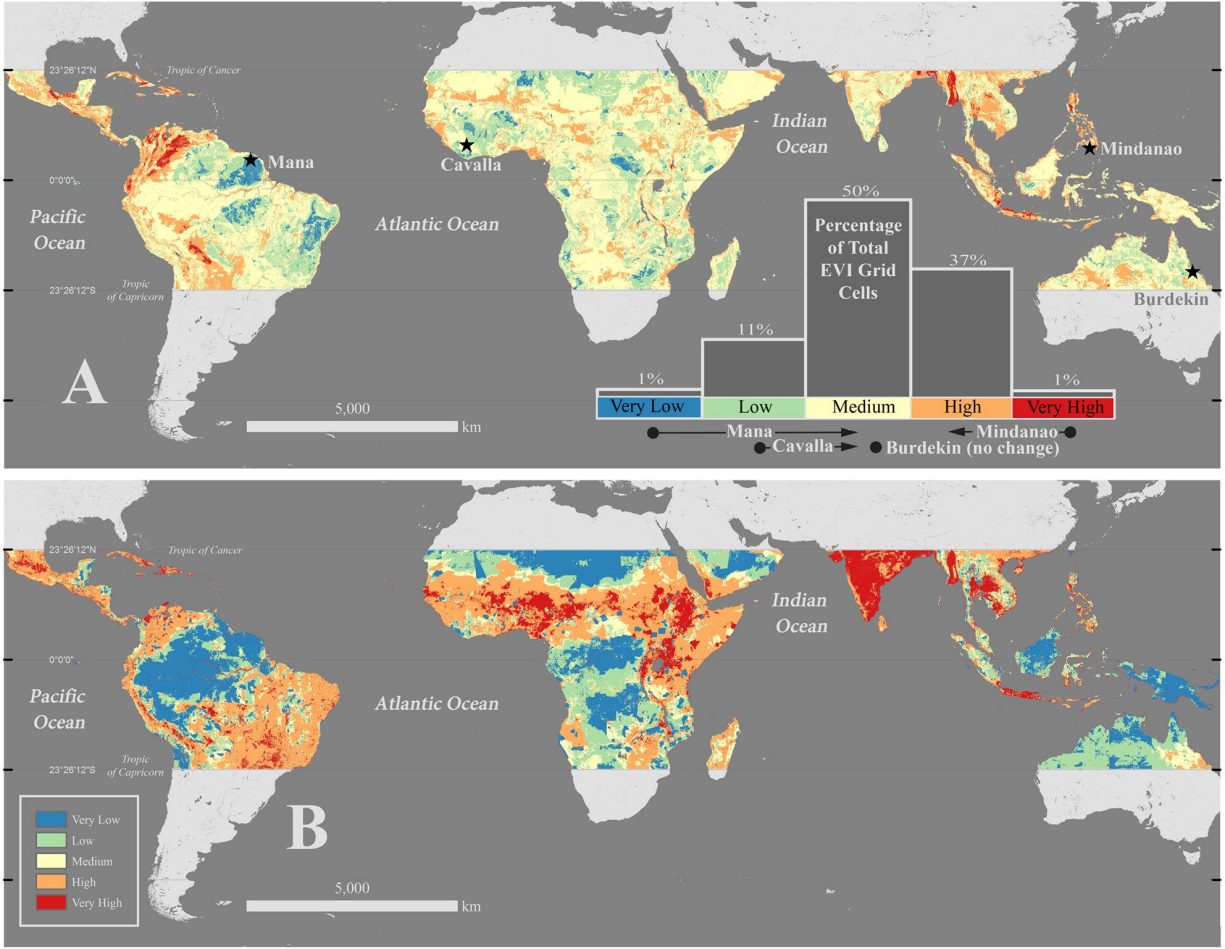
Erosion Vulnerability Index (EVI) (**A**) and Agriculture, Grazing, Mining, and Development Risk Factors (**B**) in the tropics. **A** Erosion Vulnerability Index (EVI) in the tropics (23.5° N–23.5° S) with histogram distribution of EVI and locations of 4 case studies investigated. High Risk Categories occur throughout most of Southeast Asia, along the northern Andes, in the Caribbean and through most of Central America. Low Risk Categories occur in the lower Amazon watershed and South Brazil with Low and Very Low Risk Categories throughout Africa. Histogram plot shows the entire EVI distribution over the tropics. Below the histogram plot are the names of the watersheds investigated as case studies, arrows represent how the vulnerability changed from EVI to EDVI. **B** The Risk Factor distribution for the Agriculture, Grazing, Mining, and Development (AGMD) variable. Note the relative lack of development throughout the tropics isolated primarily to the following regions: central and eastern Australia, central Africa, New Guinea, Borneo, and the Amazon River watershed. With impending development in the tropics this indicates that erosion issues could worsen if these undeveloped areas become developed. India is shown here almost completely covered by Very High Risk Factor for AGMD (**B**) though in **A** the EVI is reflected primarily in the Medium Vulnerability Class. This is due to half the Risk Factors in India were either Low Risk Factor (Earthquake Intensity Probability, Bedrock Lithology) or Very Low Risk Factor (Mean Watershed Slope, Soil Thickness) and very uniform in coverage. These figures were created using ESRI ArcDesktop 10.6, www.esri.com.

The generated EVI spans all terrestrial areas in the tropical band (23.5° N to 23.5° S) in a total number of 8.86 × 10^8^ grid cells at a resolution of 0.0004° × 0.0004° (~ 45 × 45 m) (Table 1, Supplemental Table S1). Approximately 50% of all grid cells in the tropics are in the Medium Risk Category, while 37% are in the High Risk Category, and 11% are in the Low Risk Category (Fig. 1a). The end member Risk Categories of Very High and Very Low combined makeup less than 2% of EVI grid cells (Fig. 1a, Table 2). The most common locations for Very High and High Risk Categories are in Southeast Asia, Central America, the Caribbean, and the northern Andes Mountains (Fig. 1a). Locations that occupy the Low and Very Low Risk Categories tend to occur in the lower Amazon River basin, eastern Brazil, and portions of central Africa (Fig. 1a).

**Table 1:**
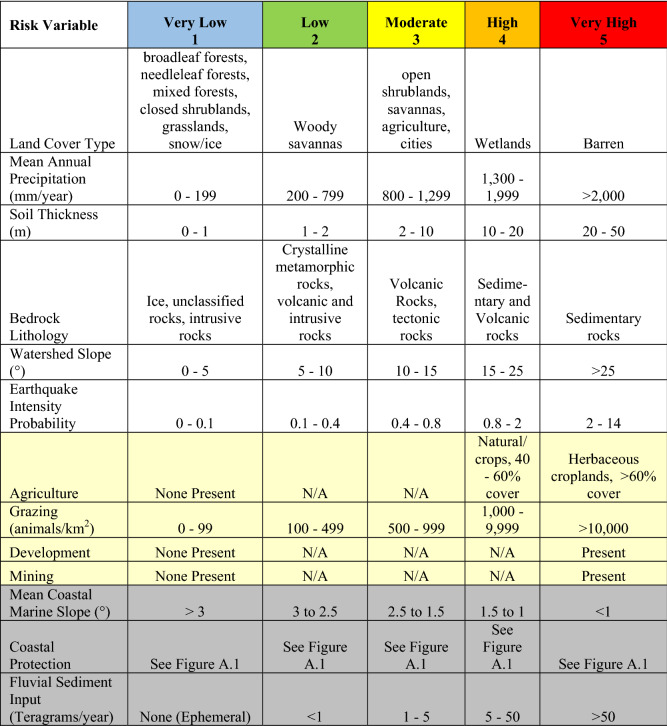
The ten variables used in Erosion Vulnerability Index (EVI) analysis (white and pale yellow cells) and the 3 Erosion and Deposition Vulnerability Index (EDVI) variables (gray cells) and their Risk Factor ranges.

Datasets values listed above are converted to Risk Factors of Very Low to Very High or numerically 1–5. Variables highlighted in pale yellow were combined into one Risk Factor for analysis (AGMD), hence the 7 Risk Factors ultimately used in the EVI. Variables highlighted in gray were added when calculating the EDVI analysis, each represent 1/6 of the total EDVI grid cells. See Supplemental Fig. S1 for Coastal Protection Risk Factor details and Supplemental Table S1 for data sources.

### Case studies: assessing watershed EVI and EDVI

EDVI analysis incorporates risk to deposition to the EVI by incorporating marine variables and allows the user to focus analysis to a single watershed. We performed EDVI analysis on four case studies chosen for their diversity in watershed size, EVI, and geographic location, coastal setting, and fluvial discharge. We analyzed the Burdekin (Australia), Cavalla (Côte d’Ivoire), Mindanao (Philippines), and Mana (French Guiana). Notably, the vulnerability of most watersheds changed as a result of adding coastal deposition variables (from EVI to EDVI, Supplemental Table S2). The change was manifested either as an increase in Vulnerability Class (e.g. Mana and Cavalla, Supplemental Table S2), or a reduction in Vulnerability Class (e.g. Mindanao, Supplemental Table S2).

**Figure 2:**
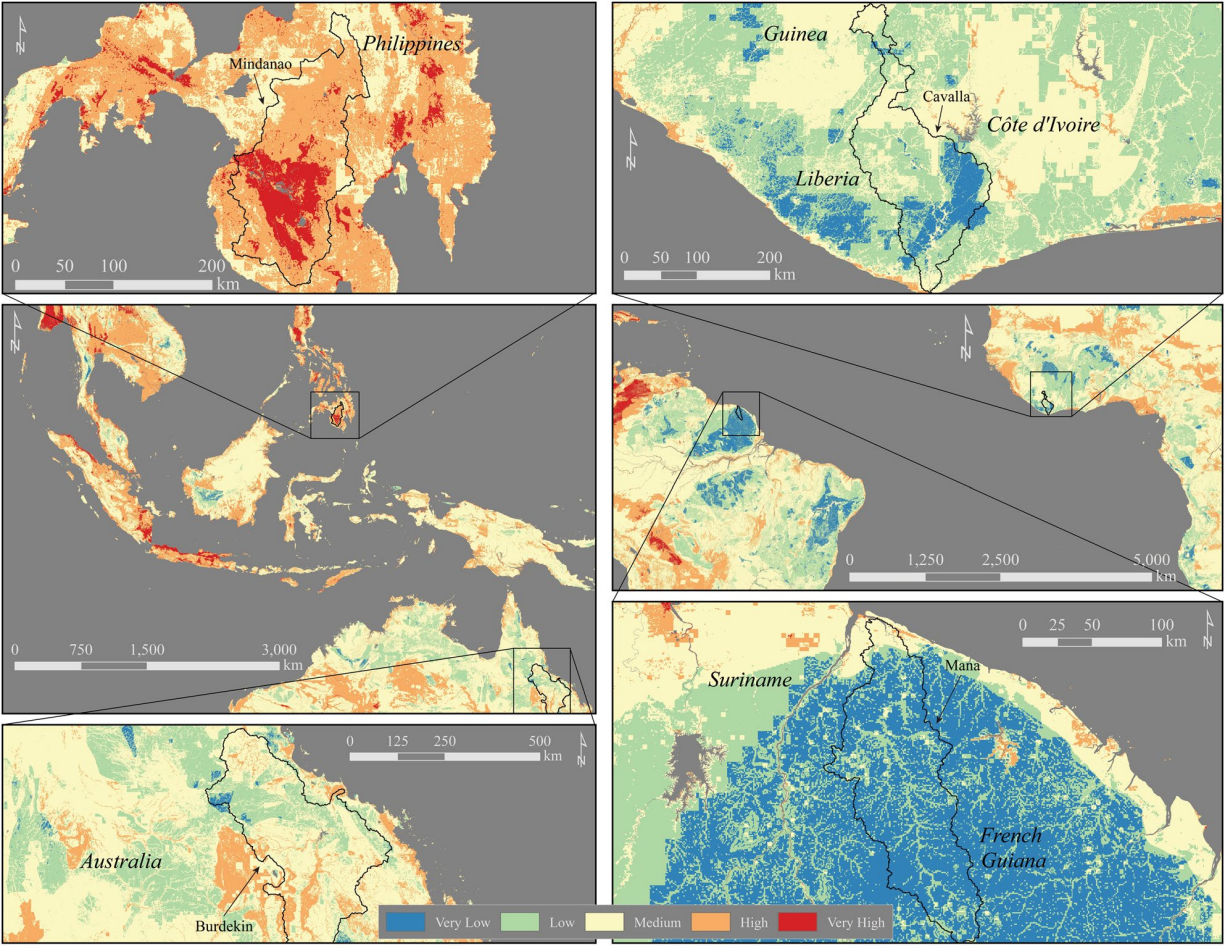
Erosion Vulnerability Index (EVI) in four case study locations. Top Left: The Mindanao watershed, Mindanao, The Republic of the Philippines, dominated by High and Very High Risk Categories. Center Left: Southeast Asia and Oceania. Bottom Left: The Burdekin watershed, Queensland, Australia, note the difference in Risk Categories from the upper watershed toward the coast. Top Right: The Cavalla watershed, Liberia, Guinea, and Côte d’Ivoire, note the prevalence of Low and Very Low Risk Categories. Center Right: Eastern South America and western Africa. Bottom Right: The Mana watershed, French Guiana, covered almost completely by Very Low Risk Categories. This figure was created using ESRI ArcDesktop 10.6, www.esri.com.

The Cavalla watershed in western Africa has a Low EVI Vulnerability Class (Fig. 2, Supplemental Table S2). However, the EDVI analysis of the Cavalla watershed results in an increase to the Medium Vulnerability Class (Figs. 1, 2, Supplemental Table S2) because of Very High Risk Factors for Mean Coastal Marine Slope and Coastal Protection (Table 1).

Unlike the Cavalla, the Mindanao watershed in the Republic of the Philippines, is an example of the EDVI analysis decreasing the Vulnerability Class (Figs. 1, 2, Supplemental Table S2). This watershed has a Very High Vulnerability as defined by the EVI. However, the EDVI decreased to High Vulnerability Class due to a Moderate Risk Factor of Coastal Protection combined with Very Low Risk Factor for Mean Marine Coastal Slope (Table 1).

The Mana watershed in French Guiana has a Very Low Vulnerability Class EVI assignment and was dominated by Very Low and Low Risk Category cells in the watershed EVI (94% of all cells) similar to the Cavalla watershed (Figs. 1, 2, Supplemental Table S2). However, the EDVI Vulnerability Class for the Mana increased 2 classes to the Medium Vulnerability Class unlike the Cavalla. This was due to the High Risk Factor Fluvial Sediment Input and Very High Risk Factor Coastal Marine Slope (Table 1, Supplemental Table S2).

The EVI in the Burdekin watershed in Queensland, Australia is an example of a watershed for which the EDVI analysis does not result in a change in Vulnerability Class. The Burdekin has a Medium Vulnerability Class EVI with the majority of the EVI grid cells in the Medium Risk Category (61% of all cells) (Fig. 2, Table 2 and Supplemental Table S2). EDVI analysis increased the risk slightly (287–327 Vulnerability Class values, Table 2) but did not change the Vulnerability Class (Supplemental Table S2). The Burdekin watershed is partially outside of the tropics and approximately 7% of the watershed is excluded (Fig. 1a). However, we do not expect this missing portion to affect the EVI analysis because even if the all the missing grid cells were exclusively Very High & High Risk Categories (unlikely given the surrounding data and existing trends) it would just barely increase the Vulnerability Class to High.

**Table 2:**
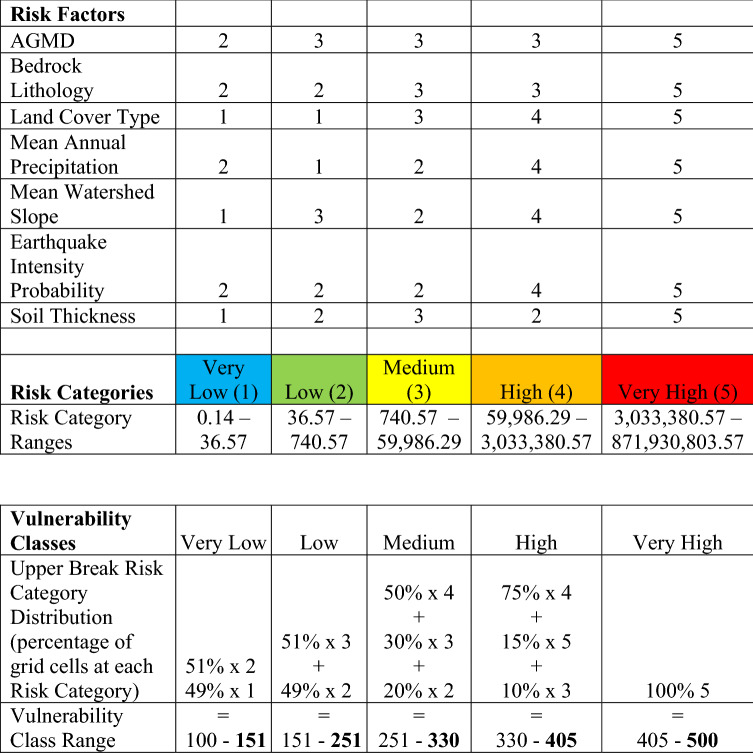
Risk Factor (RF) distribution in Risk Categories for the tropical Erosion Vulnerability Index (EVI) and Vulnerability Classes for watershed EVI and Erosion and Deposition Index (EDVI).

## Discussion

The results demonstrate that the tropics are naturally vulnerable to erosion and deposition in most regions (Fig. 1a). This is enhanced by increased land use change and development. The natural vulnerability is associated with a common environmental setting in the tropics: steep sloped watersheds with high and/or intense rainfall and a high probability of earthquakes. Connecting coastal deposition to the watershed erosion vulnerability has the ability to bring about drastic changes that both increase and decrease the vulnerability of a given connected watershed and downslope coastal zone to erosion and deposition.

### Land use change increases already risky watersheds

Tropical watersheds naturally have a high risk to erosion, which is exacerbated by land use change. Half of the grid cells in the EVI are in the Medium Vulnerability Class meaning that most areas are predisposed to risk (Fig. 1a). In the tropics, rainfall runoff drives erosion and the subsequent delivery of sediments to the downslope watershed and coastal zone. Runoff of the type infiltration-excess overland flow (when the infiltration capacity of sediments is exceeded by precipitation rate)^44^ is common in the tropics where rainfall is either seasonally sporadic, quick, and intense (high latitudes) or consistently so (low latitudes)^1^. Land use change can lower the infiltration capacity of soils, which increases runoff and erosion, by creating impermeable surfaces (urban areas) or regularly irrigating agricultural and grazing fields. This is demonstrated by Very High and High AGMD Risk Factors increasing EVI vulnerability in Central America, the Andes Mountain region, Brazil, Sub-Saharan Africa, India, Myanmar, Thailand, Cambodia, Laos, the Philippines, and Java (Fig. 1b). Although not all of these areas were classified in the High or Very High Vulnerability Class, all regions would have received a lower vulnerability class had no land use change occurred.

The relative lack of development throughout the tropics demonstrated by Fig. 1b (44% Very Low and Low Risk Factors) and the distribution of EVI grid cells shows that the vast majority (88%) of the tropics is at Medium to High Vulnerability to erosion even before considering coastal variables (Fig. 1a). Medium Vulnerability means erosion and deposition is likely and this is often preceding any major land use change activity as much of the tropics is still yet to be developed^6^. The High Vulnerability Class makes up 37% of the total EVI grid cells compared to 11% in the Low Vulnerability Class. This highlights the impact of land use change on a naturally vulnerable environmental setting i.e. high rainfall, steep watershed slope, and high probability of earthquakes as seen in Central America, the Andes, and Myanmar (Fig. 1a).

### The importance of rainfall, earthquakes, and watershed slope

The EVI identified regions with the combination of High and Very High Risk Factors for Mean Annual Precipitation, Mean Watershed Slope, Earthquake Intensity Probability, and Soil Thickness as High and Very High Vulnerability Classes. Many tropical areas are on active tectonic settings that are steep and mountainous (Caribbean islands, Southeast Asian coast and islands, the Andes, the Sierra Madres, and the Eastern Rift Mountains) leading to high weathering rates^8,10,45^. Thus, most of these regions are dominated by High and Very High Risk factors with the exception of the Eastern Rift Mountains (Fig. 1a). This result is logical given that high rainfall often drives high runoff in the tropics and this coupled with high steep slopes and earthquakes, which can induce landslides, leads to high erosion risk. Additionally, these variables are uniform in coverage, meaning no abrupt changes, leaving large regions of High and Very High Vulnerability Classes in these regions with almost no Very Low and Low Vulnerability Classes (Fig. 1a). We hypothesized that regions such as the Caribbean, the Andes, and Southeast Asia would have High and Very High Risk Categories because of the prevalence of mountainous, tectonically active, developed areas with high rainfall and thick soils. These regions are identified by our analysis (Fig. 1a) and are good validation.

### Land–ocean connections impact vulnerability

The EDVI analysis demonstrates the importance of connecting the watershed to the coastal zone to fully assess vulnerability to erosion and deposition. The increase in Vulnerability Class from EVI to EDVI in the Mana and Cavalla watersheds and the decrease in the Mindanao watershed (Figs. 1, 2, Supplemental Table S2) highlights the impact that the downstream coastal system will have on overall vulnerability. In these three case studies where EDVI differed from the EVI, Mean Coastal Marine Slope (0.05°, 0.63°, and 5.30° respectively) and Fluvial Sediment Input (6.0, 1.4, and 24.27 Tg/yr, respectively) play major roles in altering the Risk Categories assignments made exclusively in the watershed (EVI). In general, watersheds with significant fluvial sediment input possessed either deltaic features or were upslope of a broad shelf increasing sediment deposition probability. The Mana watershed jumped 2 Risk Categories from EVI to EDVI despite a Low Risk Factor for Coastal Protection. This is justified because of the impact the Very High Risk Factor and High Risk Factor Mean Coastal Slope and Fluvial Sediment Input that will favor sediment deposition. Although the Mana EVI generally indicated Low Risk (94% of all cells were in the Very Low or Low Risk Category), Fluvial Sediment Input is a High Risk Factor and the coastal zone will likely retain most of the eroded sediment due to the low mean marine coastal slope angle.

### Benefits and limitations of the EVI-EDVI method

This GIS based method utilizes open-source datasets backed by measured field data, and is easily replicable. Given that the tropics are rapidly growing, economically and in population, this open-source approach is useful for tropical leaders and community developers that may not have access to larger more extensive datasets. This method is user-friendly and computationally simple such that it can be used by scientists and decision-makers in developing tropical countries. An advantage of this method is the elimination of model usage, specifically soil water erosion models, and the exhaustive and technical validation that accompanies it. This method was developed in ESRI ArcGIS but it can be replicated using the steps laid out in Supplemental Material using QGIS or any other open-source GIS program with minimal GIS experience required. In addition, the EVI and all components used to generate it, including the Risk Factor datasets generated, are available to the public. All raw and processed data as well as step by step instructions on how to replicate the analysis are available at https://zenodo.org/record/4544155#.YC7h7WhKgb4.

Our tropics-wide EVI and EDVI approach for analyzing erosion and deposition in the coastal zone is a first-order approximation of the current risk status in tropical watersheds. Adding locally sourced and higher resolution data, during the EVI and EDVI analysis stage, will increase the effectiveness of the prediction. Global products produced such as Yang, et al.^46^ that use a more modeled approach (RUSLE method or other Soil Water Erosion Models, SWEM) incorporate important factors such as soil type and rainfall intensity. However, these modeled datasets tend to be less accurate in the variable tropics, despite improvements^42^. SWEM models such as RUSLE are useful but are less effective when applied globally and results can vary significantly regionally^47^. In addition, models require an experienced researcher to run and constrain. By utilizing datasets that do not rely on modeled analysis, our product is more approachable to non-technical users. Variables that are unrealistic to input or have yet to be properly modeled at this scale such as tidal range, wave action, and soil type would further improve the analysis. Small islands will be an area of focus in future research to assist those regions with a global data scarcity. We suggest that when analyzing islands under 1000 km^2^ locally sourced data should be used. Finally, the EVI and EDVI are similar to most large-scale vulnerability indices in that they are limited by data availability and resolution, which ultimately hinders their ability to be validated^5,30^.

The EVI and EDVI highlights the connected nature of watersheds and their connected aquatic ecosystems that underpin emerging tropical economies, and render critical ecosystem services such as tourism, coastal protection, and drinking water. Developers, policy makers, and concerned citizens, can all use the EVI/EDVI system to better understand potential consequences and identify less vulnerable regions for development. Scientists and researchers can utilize the EVI/EDVI system to identify ecosystems that might currently be at risk or regions that may be at risk in the future.

However, higher-resolution global datasets are needed to validate and improve this method, including terrestrial sediment yield and marine deposition rate data. While this is a large undertaking, efforts could begin by archiving site-specific data in a single, open source location. This would allow for limited validation which could be applied more broadly to similar sites across the globe. A united push in the international community to archive site-specific data would facilitate important validation efforts. In addition, interdisciplinary conferences are important to foster coordination and community alignment on focus areas and/or datasets. The work presented here was inspired by collaborating with researchers from many disciplines and through American Geophysical Union’s collaborative Chapman Conference in 2016 on Emerging Issues in Tropical Ecohydrology^48^.

## Methods

### Rationale for separate indices: the EVI and EDVI

The goal of this work was to create a single vulnerability index of watershed erosion and coastal deposition that could be applied anywhere in the tropics. Critically, this would connect watershed erosion and coastal deposition on a global scale, something that has yet to be accomplished to our knowledge. To assess the complex processes involved in sediment erosion and deposition, simplifying assumptions were necessary to make this tractable at a global scale. To accomplish this, we generated the Coastal Protection and Coastal Slope variables. However, both variables had to be calculated on a case by case or site-specific basis due to the complexity of coastline morphology. Thus, we were unable to create a single index for the entire tropics. Future work will attempt to accomplish this.

Instead we chose to separate the watershed erosion component into its own index (EVI) with data covering the entirety of the tropics (Fig. 1a). The combined watershed erosion and coastal deposition index (EDVI) is then computed as an extension of the EVI on the site-specific scale. The EVI represents erosion vulnerability in the watershed across the entirety of the tropics. It assumes nothing about watershed connectivity, flow accumulation, sediment delivery to the coast, or coastal deposition because those variables are not available at the global scale. The EDVI combines the EVI assessment for a site-specific watershed with 3 marine variables that consider coastal deposition. Below we detail the creation and rationale for both the EVI and EDVI.

### Layout and rationale of the EVI

We considered the following environmental and anthropogenic factors in our assessment of erosional vulnerability: Mean Annual Precipitation, Land Cover Type, Mean Watershed Slope, Bedrock Lithology, Earthquake Intensity Probability, Soil Thickness, Agriculture, Grazing, Mining, and Development. We choose these factors based on the following: 1. various Vulnerability Indices^28,30,41,49–53^ 2. Soil Water Erosion Models (SWEM) such as the Revised Universal Soil Loss equation^54^ and others e.g.^5,55^, 3. computational limitations, and 4. data availability at the global scale. Though there are many factors that contribute to sediment transport, erosion, and deposition we are limited by global data availability and resolution. After a completing a watershed scale analysis (EVI), site specific data can be considered to clarify the understanding of erosion and deposition within that catchment (as demonstrated in our case studies). At its current stage, the EVI is only a first approximation of the vulnerability erosion in all tropical watersheds.

The EVI formulation is modeled in a manner similar to the NASA and USGS coastal vulnerability indices^30,49^ using the mean product of squares of the variables chosen (Table 1). The mean product of squares formula was chosen to minimize the impact of each individual variable, in the event of misclassification errors that would be expected for relatively coarse global datasets.

The EVI includes 7 variables: Land Cover Type, Mean Watershed Slope, Bedrock Lithology, Soil Thickness, Mean Annual Precipitation, Earthquake Intensity Probability (10% chance of minimum peak ground acceleration in the next 50 years), and a combined variable, denoted AGMD, consisting of anthropogenic factors of agriculture, grazing, mining, and development (Table 1). Each of the 7 variables were classified into Risk Factors and divided into five classes from 1 (Very Low) to 5 (Very High) (Table 1). These classification breaks in Table 1 were determined based on the histogram distribution of each individual dataset, and when appropriate, other published vulnerability indices e.g.^28,30,40,41,49–55^ and many others. Below is the class break rationale for variables where breaks could not be determined by histogram distribution and published indices.

Land Cover Type: We establish these conditions under the following well established assumptions. Vegetation reduces erosion through: leaves hindering rainfall intensity, vegetation absorbing water to prevent larger peak flows, roots stabilizing the soil, water being the dominant erosive force in many environments^56,57^. We began by establishing the extreme end members with presence or absence of sediment binding roots. Those without roots (vegetation) have the highest Risk Factor (Very High or 5). Constant presence of water (wetlands) will serve to create more regular erosion than regions where it is rainfall dominant (High or 4 Risk Factor). Root depth and ground coverage are the next factors we considered. Vegetation with a shallow root system (shrublands, savannas, agriculture) may be susceptible to upheaval and subsequent erosion (Moderate or 3 Risk Factor). Those with large consistent ground cover (forests and grasslands) are more protected from rainfall intensity (Low and Very Low Risk Factors) compared to open shrublands, savannas, and agriculture which are more sparsely vegetated (Moderate or 3 Risk Factor).

Bedrock Lithology: We generate the class breaks for this variable using these general assumptions about rocks. Sedimentary and volcanic rocks are less consolidated (given that they are usually composed of pieces of other rocks) than tectonically altered rocks^58^. Tectonically altered rocks are less stable than crystalline metamorphic and intrusive igneous rocks given that they have likely been subjected to faulting or folding^58^.

Agriculture, Grazing, Mining, and Development (AGMD): Agriculture enhances erosion in many cases due to a variety of reason including loose soils, weak root structures, linear planting, monoculture and weeding, as well as consistent saturation or oversaturation of the soils^56^. Thus, greater crop cover receives a Very High or 5 Risk Factor while lesser receives a High or 4 Risk Factor. The Mining and Development variables are binary (e.g. there is either a mine or structure in grid cell or there is not). Most mines and structures have both been well documented for their contributions to erosion^56^ therefore are Very High or 5 Risk Factor for their presence. Thus, all human development variables were combined and denoted Agriculture, Grazing (sheep, horses, goats, cattle, & buffaloes/ km^2^), Mining, and Development (AGMD). This was done to consider multiple human development variables while reducing their overall impact and because the binary variables would skew the results of the EVI if accounted for individually (Supplemental Table S1).

### Execution and creation of the EVI

The EVI and all Risk Factors are calculated grid cell by grid cell (a single cell is 463 × 463 m). The 7 Risk Factors each have an individual value at a single grid cell, an integer ranging from 1 to 5. Those integers are entered into Eq. (1):

1 $$EVI={(LC}^{2}* {AGMD}^{2}*{L}^{2}*{ST}^{2}*{P}^{2}*{WS}^{2}*{EQ}^{2})/7$$

where LC is Land Cover Type, AGMD is Agriculture, Grazing, Mining and Development, L is Bedrock Lithology, ST is Soil Thickness, P is Mean Annual Precipitation, WS is Mean Watershed Slope, and EQ is Earthquake Intensity Probability. Equation 1 is adapted from the NASA Coastal Vulnerability Index^49^ developed for the coastal United States. Gornitz and White^49^ and Gornitz, et al.^59^ experimented with several formula types and ultimately determined that the form of Eq. (1) accurately represented the data while diminishing the contribution of a single variable to prevent misclassification errors. This is advantageous and we also adopt this equation given that coarse global datasets are likely to have some errors or misrepresentations. The details of how each raster dataset was processed can be found in the Supplemental Material.

EVI values from Eq. (1) can range from a minimum of 0.14 (all Risk Factors having a value of 1) to a maximum of 8.7 × 10^8^ (all Risk Factors having a value of 5) (Table 2). The total distribution of calculated EVI values was divided into *Risk Categories*: Very Low 0.14–36.57, Low 36.57–740.57, Medium 740.57–5.9 × 10^4^, High 5.9 × 10^4^–3.0 × 10^6^, and Very High 3.0 × 10^6^–8.7 × 10^8^ (Table 2). We consider a grid cell composed solely of 1 and 2 Risk Factors as Very Low (Table 2). We consider Very High as composed of all 5 Risk Factors (Table 2). In between these two end members our methodology specified that the high breaks for each class will include at most 4 Risk Factors (> 50%) of that class or a higher class (e.g. a grid cell in the Medium Risk Category will include at most four 3 s) (Table 2). Results are displayed on a map with a color assigned to each grid cell according to the Risk Category of the cell (Fig. 1a). Risk Categories represent the class breaks chosen to represent and individual grid cell of the EVI as shown in Fig. 1a, class breaks are shown in Table 2.

### Layout and rationale for the EDVI

As mentioned above the EVI only considers watershed erosion thus we created an extension of the EVI the Erosion and Deposition Vulnerability Index (EDVI) which combines the EVI analysis with variables that assess coastal deposition variables. The EDVI must be completed for a single watershed and coastal zone due to the complexity of coastline morphology that is considered while assessing coastal deposition. The EDVI combines marine deposition to the watershed EVI with the addition of 3 new variables, Coastal Protection, Mean Coastal Marine Slope, and Fluvial Sediment Input (Table 1). At this time, we do not consider tidal ranges, wave heights, sea level rise, or wind effects.

Coastal Protection is determined using satellite imagery (Supplemental Table S1) to establish locations of the coastline and outlet (Supplemental Fig. 1). A coastline is defined as the interface between seawater and the land. A river or watershed outlet is defined as the point at which freshwater discharges to the ocean. After determining the distance and path from the watershed outlet to the coastline Supplemental Fig. 1 is used to estimate how protected the outlet is and if watershed sediments are at risk of deposition in the coastal zone. Distances and morphologies for each class break that represent Risk Factors for this variable were developed by observing numerous watershed outlets and coastlines and establishing a histogram distribution for coastline length and morphology. Ranges were then established to approximate the vulnerability to trapping greater amounts of sediment. This is a first order approximation of the affect of coastal protection on a sediment deposition. Mean Coastal Marine Slope was determined by drawing a 10 km transect perpendicular to the coastline from the outlet seaward. The mean slope of this transect was calculated to obtain the Mean Coastal Marine Slope for any watershed. Fluvial Sediment Input was obtained by utilizing the ISLSCP Global River Flux dataset^60^ generated by NASA (Supplemental Table S1). This data is coarse in resolution (0.5°) and local sediment discharge measurements should be used if possible. The marine EDVI variables were converted to Risk Factors (Table 1) in the same manner as the EVI.

The EDVI can only be used on the watershed scale. Thus, the EVI data covering the watershed of interest is separated from the tropics wide EVI raster dataset shown in Fig. 1a. Then, the grid cells for each Risk Category are summed and broken into percentages (e.g. 20% of the watershed is made up of Risk Category 1, 10% of Risk Category 2, 70% of Risk Category 4). The percentages are multiplied by their Risk Category Numeral (e.g. 20% × 1; 10% × 2; 70% × 4) (Table 2). This yields a range of values we term Vulnerability Classes (Table 2). The low end of the range is 100 (100% x Risk Category 1) and the high end of the range is 500 (100% x Risk Category 5) (Table 2). Vulnerability Class breaks from Table 2 mimic the breaks chosen for the Risk Categories.

The watershed of interest will then be assigned a Vulnerability Class according to the percentage of Risk Category grid cells it possesses. The EDVI is then the Vulnerability Class of the watershed specific EVI plus the 3 marine deposition variables mentioned above. Both the watershed specific EVI and marine deposition variables are considered equally (i.e. half of the grid cells represent the watershed specific EVI and half represent the new marine deposition variable Risk Categories). The EDVI is then displayed on the same Vulnerability Class scale outlined above (Table 2). Step by step instructions of this process are available in the Supplemental Material. We describe the calculation of the EVI and EDVI in detail with an example in the following section.

### Execution and creation of watershed EVI and the EDVI

For additional details including step-by-step instructions for EVI and EDVI analysis in any GIS interface, the reader is referred to Supplemental Material. To calculate watershed EVI, the total percentage of grid cells in each Risk Category were multiplied by the Risk Category numeral (Very Low–Very High or 1–5) to establish Risk Category Values. For example Watershed A has a total of 100 grid cells, 25 are in the Low Risk Category, 25 are in the Medium Risk Category, and 50 are in the High Risk Category. The total percentage of grid cells in each Risk Category in Watershed A are as follows: Low: 25%, Medium: 25%, High: 50%. These percentages are multiplied by their Risk Category Numerals, Low: (25*2) = 50, Medium: (25*3) = 75, High: (50*4) = *200, to obtain Risk Category Values.* These values were summed and broken into Vulnerability Classes (Very Low–Very High or 0–500) which represent the EVI of the watershed (Table 2) *or for Watershed A: 50* + *75* + *200* = *325, so Watershed A has an EVI of Medium* (Table 2).

To balance the contribution of marine and watershed based Risk Factors, the same number of total grid cells that represent the watershed EVI (*100 for Watershed A)* also represent the 3 marine EDVI Risk Factors. Thus the 3 EDVI Risk Factors each compose 1/6 of the overall EDVI while the watershed EVI makes up the other half of the cells. *Continuing the example above with Watershed A which, had a total of 100 grid cells; the total grid cells for the overall EDVI is 200, 100 of which make up the original EVI and 100 new cells distributed evenly between the 3 new marine EDVI Risk Factors (33.33 cells each).* Each EDVI variable is assigned a single Risk Factor (Very Low–Very High or 1–5) to represent all its cells, based on it Risk Factor classification as shown in Table 1.

The percentage distribution of Risk Factors was recalculated with the new total grid cells to include the 3 new EDVI Risk Factors. *Continuing the Watershed A example, Coastal Protection is Very High (Very High Risk Factor or 5), Fluvial Sediment Input is less than 1 Tg/yr (Low Risk Factor or 2), and Mean Coastal Marine Slope is 2.7° (Low Risk Factor or 2). The EDVI Risk Factor cells then become: Low Risk: 66.66 and Very High Risk: 33.33. These are then added to the original EVI Risk Factor cells to create new EDVI Risk Category Percentages which are now: Low: 45.83%* = *((25* + 66.66)/200)*100, Medium: 12.5% = *(25* + 0/200)*100, High: 25% = *(50* + 0/200)*100, Very High: 16.665% = *(0* + 33.33/200)*100. New Risk Category Values are then generated in the same manner as the Watershed EVI and the values are summed. Risk Category Values for Watershed A are, Low: (45.83*2) = 91.66, Medium: (12.5*3) = 37.5, High: (25*4) = 100, Very High: (16.665*5) = *83.325. The EDVI of Watershed A is 91.66* + *37.5* + *100* + *83.325* = *312.485 or Medium Vulnerability Class* (Table 2)*.* The EDVI Vulnerability Classes are on the same scale as the EVI Vulnerability Classes (Table 2).

In order to obtain a wide variety of results in our analyses, we targeted large and small watersheds from each major continent and regions with a broad range of EVI values. We calculate the EVI for an individual watershed to accurately represent the distribution of Risk Categories within in that watershed. Where possible, it is desirable to use locally collected and/or higher resolution data as it will likely be more accurate than the global datasets. If no such data exist, the current EVI and the open-source datasets used for each EDVI will allow for analysis of any watershed in the tropics.

## Supplementary information

Supplementary Information
