# Author Correction: Vulnerability to watershed erosion and coastal deposition in the tropics

**DOI:** 10.1038/s41598-021-94537-2

**Published:** 2021-07-21

**Authors:** Trevor N. Browning, Derek E. Sawyer

**Affiliations:** School of Earth Sciences, The Ohio State University, 125 South Oval Mall, Columbus, OH 43214 USA

Correction to: *Scientific Reports* 10.1038/s41598-020-79402-y, published online 13 January 2021

The original version of this Article contained an error.

In the Discussion section, under the subheading “Benefits and limitations of the EVI‑EDVI method”,

“All raw and processed data as well as step by step instructions on how to replicate the analysis are available on pangaea.de”

now reads:

“All raw and processed data as well as step by step instructions on how to replicate the analysis are available at https://zenodo.org/record/4544155#.YC7h7WhKgb4”.

The original Article has been corrected.

