# Supplementary material for: Vulnerability to watershed erosion and coastal deposition in the tropics
Source: Sci Rep. 2021 Jan 13;11:885. doi: 10.1038/s41598-020-79402-y (PMC7806860; doi:10.1038/s41598-020-79402-y)
Supplement: Supplementary file 1 — Supplementary Information [file 41598_2020_79402_MOESM1_ESM.pdf]

Supplemental Information for:

Vulnerability to Watershed Erosion and Coastal Deposition in the Tropics

Trevor N. Browning* and Derek E. Sawyer

The Ohio State University
School of Earth Sciences
125 South Oval Mall
Columbus, OH 43214 USA

*Corresponding author: browning.257@osu.edu

Introduction

The following Supplemental Material contains: detailed methods and rationale behind the creation of the EVI and EDVI, step-by-step instructions to generate all EVI and EDVI files in ArcGIS as well as the EVI and EDVI of any watershed in the tropics, a figure describing how to calculate the Coastal Protection variable, a table with detailed information regarding all used variables, and a table demonstrating characteristics of the 4 case studies chosen.

Supplemental Methods

Several erosion triggers/enhancers either did not exist at a global resolution (e.g. tidal range, watershed connectivity, and various human development activities) or are modeled products with questionable accuracy (e.g. rainfall erosivity, presence of dams or other engineering structures, and soil type) and were not included in the EVI. Accurate tidal range at this scale was not possible and is a lesser driver of erosion in most cases when compared to watershed erosion. To account for human infrastructure we used a dataset called Global Urban Footprint (Esch et al., 2018) developed by the German Aerospace Center (DLR) at their Earth Observation Center. Global Urban Footprint identifies 'settlements', not roads or other similar transportation infrastructure.

Of the 10 EVI variables (ultimately 7 Risk Factors), 8 global raster datasets were available and the remaining 2 were in vector format (point or polygon data) (Supplemental Table 1). All datasets were downloaded from open access servers and are free for public use (Supplemental Table 1). The point-based vector dataset (mining) was rasterized at a resolution of 0.01° (Supplemental Table 1). The polygon-based vector dataset (bedrock lithology) was rasterized at the same resolution downloaded (0.67° x 0.67°) (Supplemental Table 1). All rasters were clipped to the tropical band (23.5° N to 23.5° S) and converted to a geographic projection system (GCS WGS84) before analysis. The values for each variable were reclassified to Risk Factors to demonstrate erosional risk with values ranging from 1 to 5 (Table 1). Risk Factor rasters were resampled using the nearest neighbor method (no new cells generated) to 0.004° x 0.004° grid cell resolution. Land-based datasets (land cover type, AGMD, and bedrock lithology) lacked data over the ocean and had a coarser resolution along the coastline as a result. These Risk Factors' null values were reclassified to Very High Risk Factor thus changing all

cells over the ocean, and some on the coastline, to Very High Risk Factor. The coastline generally lacks vegetation and is higher energy, making it prone to erosion, hence the Very High Risk Factor assignment. This does not create much false data because of the high resolution of the three datasets (Supplemental Table 1). The combined AGMD Risk Factor raster was created by taking the maximum value at a given grid cell from each of the 4 contributing rasters. This was done to limit the individual contributions of each variable.

Step by Step Instructions to generate the EVI or EDVI

Step by Step Instructions to generate a watershed EDVI from EVI values.

ArcGIS 10.6 and earlier

1. In ArcDesktop click “Add Data” (yellow square with a black cross in it) and add the EVI raster (‘ev0004xsl7-4L’), the Fluvial Sediment Input Risk Factor raster (oc_flx_tss_rf), and the Mean Marine Coastal Slope raster (‘g19slope’), and the appropriate continent of HydroSHEDS (‘af_bas_15s_beta.shp’, ‘as_bas_15s_beta.shp’, ‘au_bas_15s_beta.shp’, ‘ca_bas_15s_beta.shp’, ‘eu_bas_15s_beta.shp’, ‘na_bas_15s_beta.shp’, ‘sa_bas_15s_beta.shp’)
    a. Find your watershed using hydroSHEDS (Lehner et al., 2008).
2. Open ArcCatalog and create a new shapefile by right clicking then select “New” and “Shapefile”
    a. Name the file the name of your watershed (e.g. exwtshd.shp), select “polygon” for format.

    b. Click the “edit” button and choose a geographic WGS84 projection for the coordinate system. Then click “ok”, and close ArcCatalog.

3. In ArcDesktop add the newly created shapefile in this example ‘exwtshd.shp’
4. Using the editor toolbar click the “editor” button and click “start editing”
    a. A dialog box will appear, select ‘exwtshd.shp’ and click “ok”.
    b. Ensure that you are in Data View (small icons on the bottom left hand corner of the map) and select your watershed of interest in the HydroSHEDS shapefile.
    c. Copy and Paste your watershed, it will prompt as to which layer to paste into and ensure that it is your new shapefile ‘exwtshd.shp’.
    d. Click “Editor” again and select “stop editing” and click “yes” to save your edits.
5. In ArcGIS use the ‘Clip’ tool to clip the EVI raster (‘ev0004xsl7-4L’)
    a. In “Arc Toolbox” it is under “Data Management” → “Raster” → “Raster Processing” → “Clip”
    b. A dialog box will appear, for “Input Raster” select the EVI raster (‘ev0004xsl7-4L’).
    c. For “Output Extent” select ‘exwtshd.shp’.
    d. Check the box for “Use Input Features for Clipping Geometry”.
    e. Use “Output Raster Dataset” to specify the location and name for the new file (e.g. exwtshdevi).
    f. Check the box for “Maintain Clipping Extent” and click “ok”.
6. Turn the new raster into integer. Use the ‘Int’ tool

a. In “Arc Toolbox” it is under “Spatial Analyst” → “Math” → “Int”
b. A dialog box will appear, for “Input Raster” select ‘exwtshdevi’.
c. Use “Output Raster” to specify the location and name for the new file (e.g. int_exwtshdevi) and Click “ok”.

7. Generate and export the arc table to an excel table
   a. Right click on ‘int_exwtshdevi’ and click “open attribute table”.
   b. Click the top left icon and click “export” in the dropdown box.
   c. For “Export” choose “all records”.
   d. Use “Output Raster Table” to specify the location and name for the new file (e.g. exwtshdtbl.dbf) and click “ok”.
   e. Use the “Table to Excel” tool to convert the .dbf to an excel table.
      i. For “Input Table” choose ‘exwtshdtbl.dbf’
      ii. Use “Output Excel File” to specify the location and name for the new file (e.g. exwtshdevitbl.xls) and Click “ok”.
8. In Microsoft Excel open your exported excel table ‘exwtshdevitbl.xls’

   Within your exported excel table

   a. ‘exwtshdevitbl.xls’ should have two data columns (A & B) titled ‘VALUE’ & ‘COUNT’
      i. The ‘VALUE’ column contains the Risk Category values for your watershed
      ii. The ‘COUNT’ column contains the amount of grid cells at that Risk Category value

b. Copy columns C-H from ‘EDVI_testwtshd_ex.xlsx’ and paste into columns C-H in ‘exwtshdevitbl.xlsx’.

c. Two cells below the last row of real data (B96 in ‘EDVI_testwtshd_ex.xlsx’) write the following

    i. = sum(B2:B**94**)

    ii. Use the last row of real data in place of B94!

    iii. This should sum all the cells in the ‘COUNT’ column. This is the total grid cells in your rasterized watershed ‘exwtshdevi’.

d. All cells that are highlighted in orange need to be changed.

    i. As you make changes it is helpful to unhighlight them to keep track.

e. Resize the cell ranges within the formulas contained in cells C2, C5, C8, C11, C14 to the proper Risk Category breaks shown in Supplemental Table 2.

f. Modify the formulas in cells E2, E3, E5, E6, E8, E9, E11, E12, E14, E15, F21, F22, F23.

    i. Change the ‘B96’ portion only and replace with your summed total grid cells cell location. Note it is B96 in ‘EDVI_testwtshd_ex.xlsx’.

g. The Watershed EVI value should have updated properly in cells G17 and H17

i. G17 represent the Vulnerability Class Value and H17 represents the Vulnerability Class; 1 = Very Low, 2 = Low, 3 = Medium, 4 = High, 5 = Very High

ii. Note that the Watershed and Coastal EDVI have NOT been calculated yet

9. In ArcDesktop zoom to your watershed and turn on 'oc_flx_tss_rf'
   a. The number at the coastal zone downslope of your watershed is the Risk Factor for Fluvial Sediment Input
      i. Input that number (1-5) in your excel sheet in cell E22.
   b. **OPTIONAL**: the raster 'ocean_flux_tss_hd_map.asc' can be used to determine the actual fluvial input and that number can be input in cell D22 at this point E22 will calculate automatically.
10. Using Supplemental Figure 1 determine your Coastal Protection Risk Factor and input into cells D23 and E23.
    a. Add satellite imagery basemap in ArcDesktop and use the "Measure" button (small ruler icon) to determine lengths, etc.
11. In ArcDesktop use the "Measure" button to draw a line perpendicular from your watershed outlet to 10 kilometers from your previously determined coastline.
    a. Tip: Using the draw toolbar draw this line and then adjust it to 10 km.
    b. Using the 3D Analyst Toolbar select the Mean Coastal Slope raster ('g19slope') and click the "Interpolate Line" button (green square with a zig-zag line above it).
       i. Draw a line with two points just beside your line.

ii. Click the “Profile Graph” button (button with a graph on it)

iii. This will generate a profile of your slope. Right click the profile and click “export”

iv. Select the “Data” tab and click “preview”. Copy the data and paste it into a **new sheet** in your excel table.

c. In Excel write step copy the following step and paste it two cells below the last cell with data in the B Column in your excel sheet

i. =average(B2:B25)

ii. **NOTE**: B25 is where our data ends in the example given adjust to the end of your real data.

iii. Write the value generated in cell D21 in your main sheet.

12. The EDVI value is now calculated properly in G18 and H18.

i. G18 represent the Vulnerability Class Value and H18 represents the Vulnerability Class; 1 = Very Low, 2 = Low, 3 = Medium, 4 = High, 5 = Very High

Step by Step Instructions to generate a global or tropical or watershed EVI. For each individual EVI variable follow these steps.

1. Use the Project tool to reproject the raster to a constant geographic projection.
   a. In “Arc Toolbox” “Data Management” → “Projections and Transformations” → “Project”.
   b. For “Input Dataset or Feature Class” select the target raster
   c. For “Output Coordinate System” specify the same geographic projection for all files for our study we used ‘GCS_WGS84’.

    d. Select the file path and execute.

2. Use the Resample Tool to resample the data to at least 0.004° x 0.004°.
    a. In “Arc Toolbox” “Data Management” → “Raster” → “Raster Processing” → “Resample”.
    b. For “Input Raster” select the projected raster.
    c. For “Output Cell Size” ‘X’ type in 0.004 and ‘Y’ type in 0.004
    d. For “Resampling Technique” select “Nearest”
    e. Select the file path and execute.
3. Use the “Reclassify” Tool to reclassify the values to Risk Factor values in Table 1.
    a. In “Arc Toolbox” it is under “Spatial Analyst” → “Reclass” → “Reclassify”
    b. For “Input Raster” select the reprojected and resampled raster.
    c. For “Reclassification” specify the values as those laid out in Table 1.
    d. Select the file path and execute.

To Create AGMD Risk Factor

1. Combine the maximum values from the Agriculture, Grazing, Mining, and Development Risk Factor rasters
    a. In “Arc Toolbox” “Spatial Analyst” → “Local” → “Cell Statistics”
    b. For “Input Rasters or Constant Value” select the each of the Agriculture, Grazing, Mining, and Development Risk Factor rasters.
    c. For “Overlay Statistic” select ‘Max’
    d. Ensure the box is checked for “Ignore NoData in Calculations”

e. Select the file path and execute.

Use all the Risk Factor rasters to create the tropical EVI

1. In “Arc Toolbox” “Spatial Analyst” → “Map Algebra” → “Raster Calculator”
   a. In “Map Algebra Expression” using all 7 risk factors type in a formula such as:
      i. ((("RasterA"**2)*("RasterB"**2)*("RasterB"**2)*("RasterC"**2)*("RasterD"**2)*("RasterE"**2)*("RasterF"**2))/7
      ii. NOTE: Replace RasterA-F with actual Risk Factor rasters
   b. Select the file path and execute.

Cleanup, Tips, and Subtracting Lakes

1. Subtract lakes from EVI raster
   a. Note: Depending on the resolution of the EVI raster it may need to be resampled in order to obtain higher resolution coastline data.
   b. In “Arc Toolbox” “Spatial Analyst” → “Extraction” → “Extract by Mask”
   c. For “Input Raster” select the created EVI raster file.
   d. For “Input Raster or Feature Layer” select the shapefile ‘tropics_lknull.shp’ (this was created using (ESRI, 2018)).
   e. Select the file path and execute.
2. Turn the new raster into integer. Use the ‘Int’ tool
   a. In “Arc Toolbox” it is under “Spatial Analyst” → “Math” → “Int”
   b. A dialog box will appear, for “Input Raster” select ‘exwtshdevi’.

c. Use “Output Raster” to specify the location and name for the new file (e.g. int_exwtshdevi) and Click “ok”.

d. The generated raster can be exported and develop a histogram as in Step 7 of the above *Step by Step Instructions to generate a watershed EDVI from EVI values.*

3. Tips

a. We found that resolutions above 0.004° x 0.004° would often cause errors with “Raster Calculator” or other tools while generating the EVI or even some Risk Factors. More work will be done on this in the future.

If using this Step By Step Instruction Guide please use the citation for this publication.

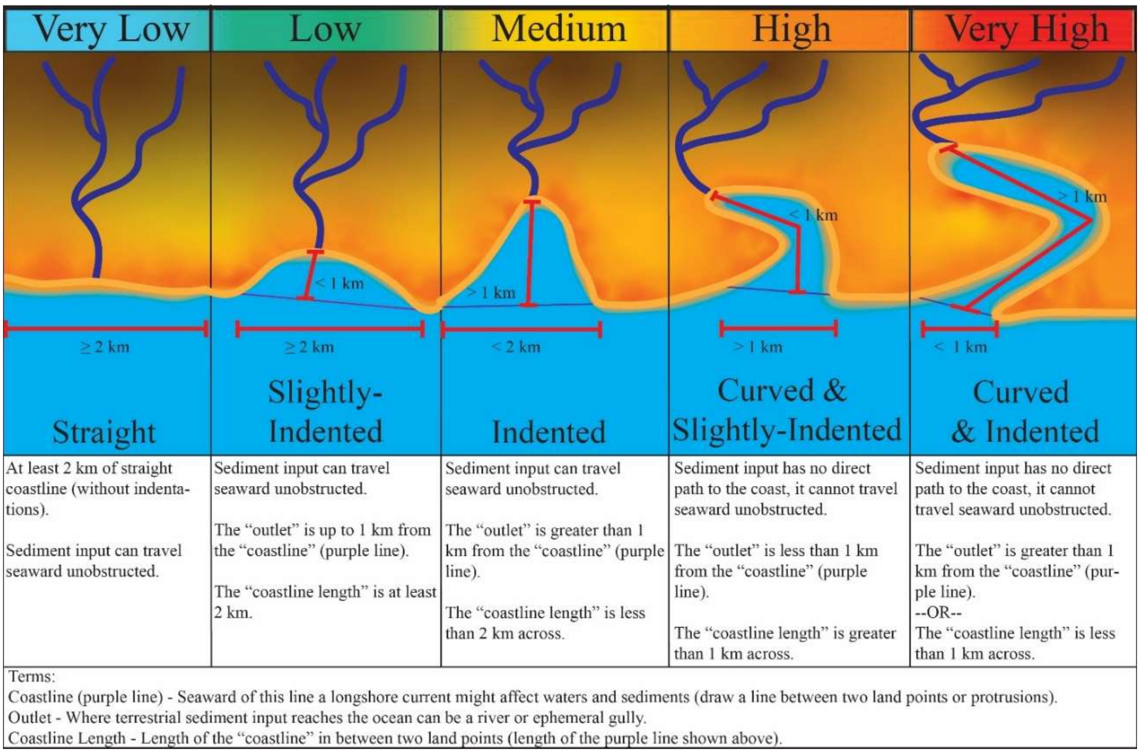

At least 2 km of straight coastline (without indentations).<br><br>Sediment input can travel seaward unobstructed.	Sediment input can travel seaward unobstructed.<br><br>The “outlet” is up to 1 km from the “coastline” (purple line).<br><br>The “coastline length” is at least 2 km.	Sediment input can travel seaward unobstructed.<br><br>The “outlet” is greater than 1 km from the “coastline” (purple line).<br><br>The “coastline length” is less than 2 km across.	Sediment input has no direct path to the coast, it cannot travel seaward unobstructed.<br><br>The “outlet” is less than 1 km from the “coastline” (purple line).<br><br>The “coastline length” is greater than 1 km across.	Sediment input has no direct path to the coast, it cannot travel seaward unobstructed.<br><br>The “outlet” is greater than 1 km from the “coastline” (purple line).<br>--OR--<br>The “coastline length” is less than 1 km across.

Terms:
Coastline (purple line) - Seaward of this line a longshore current might affect waters and sediments (draw a line between two land points or protrusions).
Outlet - Where terrestrial sediment input reaches the ocean can be a river or ephemeral gully.
Coastline Length - Length of the “coastline” in between two land points (length of the purple line shown above).

Supplemental Figure 1. Risk Factor distribution for the Coastal Protection variable. Coastlines are idealized to be compared to actual coastlines in order to categorize risk. Figure S1 was created in Adobe Illustrator www.adobe.com/products/illustrator.html

Dataset Name	Data Type	Measured by	Representative Year?	Dataset Type	Resolution (arc°)	Agency Acquired From
Land Cover Type	Land Cover Type	Satellite: MODIS	2017	Raster Grid	0.0043°	National Aeronautics and Space Administration (Friedl and Sulla-Menashe, 2015)
Mean Watershed Slope	Digital Elevation Model	Satellite and Surficial Measurements	2009 – 2018	Raster Grid	0.0042°	General Bathymetric Chart of the Ocean (GEBCO, 2019)
Soil Thickness	Average Soil Thickness	Modeled	1900 – 2015	Raster Grid	0.0083°	National Aeronautics and Space Administration (Pelletier et al., 2016)
Bedrock Lithology	Lithology	Field Surveys	N/A	Vector Polygons	0.6717°	United States Geological Survey (Chorlton, 2007)
Mean Annual Precipitation	Monthly Accumulated Precipitation	Satellite: TRMM and Surficial Measurements	2007 – 2017	Raster Grid	0.25°	National Aeronautics and Space Administration (Huffman and Bolvin, 2015)
Mining	Active Mines	Field Surveys	2003 – 2013	Vector Points	0.01°	United States Geological Survey (Schulz et al., 2018)
Development	Global Urban Footprint	Satellite: TerraSAR-X, TanDEM-X	2011 – 2012	Raster Image	0.0001°	DLR Earth Observation Center (Esch et al., 2018)
Grazing	Animals/ km^2	Census and Modeled Data	2010	Raster Grids	0.0833°	Food and Agriculture Organization of the United Nations (Gilbert et al., 2018)
Agriculture	Land Cover Type	Satellite: MODIS	2017	Raster Grid	0.0043°	National Aeronautics and Space Administration (Friedl and Sulla-Menashe, 2015)
Earthquake Intensity Probability	Peak Ground Acceleration	Surficial and Modeled Data	2000 – 2050	Raster Grid	0.1°	Helmholtz Centre Potsdam, GFZ German Research Centre for Geosciences (Giardini et al., 1999)
Mean Marine Coastal Slope	Digital Elevation Model	LiDAR and Bathymetry Measurements	2009 – 2018	Raster Grid	0.0042°	General Bathymetric Chart of the Ocean (GEBCO, 2019)
Fluvial Sediment Input	Suspended Sediment Flux	Satellite and Surficial Measurements	1986 – 1995	Raster Grid	0.5°	National Aeronautics and Space Administration (Ludwig et al., 2011)
Coastal Protection	Aerial Photos	Satellite	2015 – 2018	Image	N/A	ESRI, Google Maps

Supplemental Table 1. All 13 open source datasets used in the Erosion Vulnerability Index (EVI) Erosion and Deposition Vulnerability Index (EDVI) analysis.

Site	EVI	EDVI	EVI Value	EDVI Value	Dominant EVI Risk Category & Percent	Dominant EDVI Risk Category	Dominant EDVI Risk Category %	Region	3-D Surface Area (km^2)
Burdekin River	3	3	287.5	327.13	3; 61.36%	3	64.01%	NE Australia	120,898
Mana River	1	3	115.2	252.02	1; 67.52%	1	33.76%	NE South America	12,237
Cavalla River	2	3	212.6	322.97	2; 44.58%	3	33.61%	W Africa	29,696
Mindanao River	5	4	413.8	340.26	4; 60.38%	4	46.86%	SE Asia	23,169

Supplemental Table 2. Watershed Erosion Vulnerability Index (EVI) analysis and Erosion and Deposition Vulnerability Index (EDVI) analysis for 4 watersheds in the tropics.
